# Coordinated upregulation of PABPC1L and SNHG lncRNAs defines a tumor-specific expression module in colorectal cancer: evidence from paired tumor-normal expression profiling

**DOI:** 10.1016/j.bbrep.2025.102373

**Published:** 2025-11-20

**Authors:** Fatemeh Ghadyani, Zahra Fazeli, Solat Eslami, Mahla Sanati, Zahra Tajik, Soudeh Ghafouri-Fard, Amir Sadeghi

**Affiliations:** a Department of Medical Genetics, School of Medicine, Shahid Beheshti University of Medical Sciences, Tehran, Iran; b Department of Medical Biotechnology, School of Medicine, Alborz University of Medical Sciences, Karaj, Iran; c Non-communicable Diseases Research Center, Alborz University of Medical Sciences, Karaj, Iran; d Gastroenterology and Liver Diseases Research Center, Research Institute for Gastroenterology and Liver Diseases, Shahid Beheshti University of Medical Sciences, Tehran, Iran

**Keywords:** Colorectal cancer, RUSC1-AS1, SNHG17, PABPC1L, SNHG1, lncRNA

## Abstract

Nonsense-mediated mRNA decay (NMD) has an important role in the pathoetiology of cancer, including colorectal cancer (CRC). Identification of the lncRNAs associated with this function would enhance our understating about the molecular mechanisms of CRC and facilitate design of novel therapies. In the current study, we used a bioinformatics approach to find NMD-associated lncRNAs in CRC. Then, we assessed expression of these genes in 43 paired CRC samples and adjacent non-tumor (ANT) tissues. Three transcripts were significantly overexpressed in tumor tissue: PABPC1L (mean fold-change ≈ 5.20, 95 % CI 2.30–11.85, P = 0.0003), SNHG17 (mean fold-change ≈ 5.46, 95 % CI 2.04–14.50, P = 0.001), and SNHG1 (mean fold-change ≈ 5.82, P = 0.0086). RUSC1-AS1 showed a non-significant trend toward upregulation (fold-change ≈ 2.15, 95 % CI 0.92–5.00, P = 0.07). The combined four-gene model demonstrated moderate discriminatory power, yielding an AUC of 0.76 (95 % CI 0.65–0.86, *p* < 0.0001) with balanced sensitivity and specificity of 69.8 % each at the 0.5 cutoff (overall accuracy 69.8 %). Taken together, RUSC1-AS1, SNHG17, PABPC1L and SNHG1 can be novel candidates for future diagnostic studies in CRC.

## Highlights

• Three nonsense-mediated mRNA decay-related transcripts were overexpressed in CRC tissues.
• RUSC1-AS1, SNHG17, PABPC1L and SNHG1 represent novel candidates for diagnostic studies.
• Their dysregulation in CRC suggests their participation in its pathogenesis.

## 1 Introduction

Nonsense-mediated mRNA decay (NMD) is an evolutionarily conserved process that warrants the conformity and regulation of genes expression [1]. In fact, NMD acts as a quality-control mechanism that targets and promptly destroys aberrant transcripts that carry premature termination codons (PTCs) [2]. Besides, NMD targets several transcripts that encode functional full-length proteins, thus it serves as a mechanism of gene expression regulation [2]. In the physiological conditions, NMD has a protective role in the cell, precluding the production of probable dominant-negative proteins and decreasing the expression of several critical factors that can induce tumorigenic processes [3]. Several proteins, including Mammalian poly A-binding proteins (PABPs) are involved in the NMD [4]. These highly conserved RNA-binding proteins mainly contribute to the regulation of mRNA translation and stability. Among them is PABPC1, which is regarded as a vital modulator of cytoplasmic mRNA homing with essential roles in a magnitude of physiological and pathological processes through regulation of several aspects of RNA metabolism [5]. PABPC1 is implicated in numerous aspects of tumorigenesis as an RNA-binding protein. PABPC1 selectively regulates gene expression through interacting with long non-coding RNAs (lncRNAs) and miRNAs that increase cancer cell plasticity and promote cancer progression [5]. PABPC1L is another member of PABPC1 family of proteins that is involved in NMD and the mRNA surveillance pathway, based on Kyoto Encyclopedia of Genes and Genomes (KEGG) enrichment analysis (https://www.genome.jp/entry/hsa:80336). This study aimed to investigate the expression of lncRNAs associated with the PABPC1L gene in the NMD pathway of colorectal cancer (CRC) through assessment of their expression in paired CRC tissues and adjacent non-tumor (ANT) tissues. SNHG17, MHENCR, and RUSC1-AS1 were selected as PABPC1L-related lncRNAs through bioinformatics approach. In addition, SNHG1 was selected as an NMD-associated lncRNAs based on literature [6].

## 2 Materials and methods

### 2.1 Data acquisition and processing

The Bulk RNA Gene Expression data of 522 Colon Adenocarcinoma (COAD) samples, including 481 tumor and 41 normal samples, and 176 rectum Adenocarcinoma (READ) samples, including 166 tumor and 10 normal samples, were downloaded from The Cancer Genome Atlas (TCGA) database (https://portal.gdc.cancer.gov/). The two datasets were integrated using R software, and the batch effect was also evaluated in the final data.

### 2.2 Identification of differentially expressed genes

All raw data were preprocessed in R software to investigate differentially expressed genes (DEGs) through the DESeq2 package [7] by setting criteria with | log2FoldChange | > 1 and *P-adj*< 0.05. Next, the differentially expressed genes were categorized based on their gene type, specifically protein-coding and long non-coding RNAs.

### 2.3 KEGG and GO analysis

KEGG and Gene Ontology (GO) analyses were conducted by using the EnrichR database (https://maayanlab.cloud/Enrichr/). This step led to identification of 11 protein-coding genes that are involved in the NMD pathway (UPF1, UPF2, UPF3B, SMG1, SMG5, SMG6, SMG7, EIF4A3, MAGOH, RBM8A, and PABPC1L).

### 2.4 Spearman correlation

We computed the Spearman correlation between these NMD-related genes and the significant lncRNAs. The results were filtered by using the square of the correlation coefficient, Spearman's rho >0.4, and *P-adj* < 0.05. In summary, the findings suggest that PABPC1L was the only protein-coding gene among 11 genes that showed both significant correlation and differential expression.

**Fig. 1:**
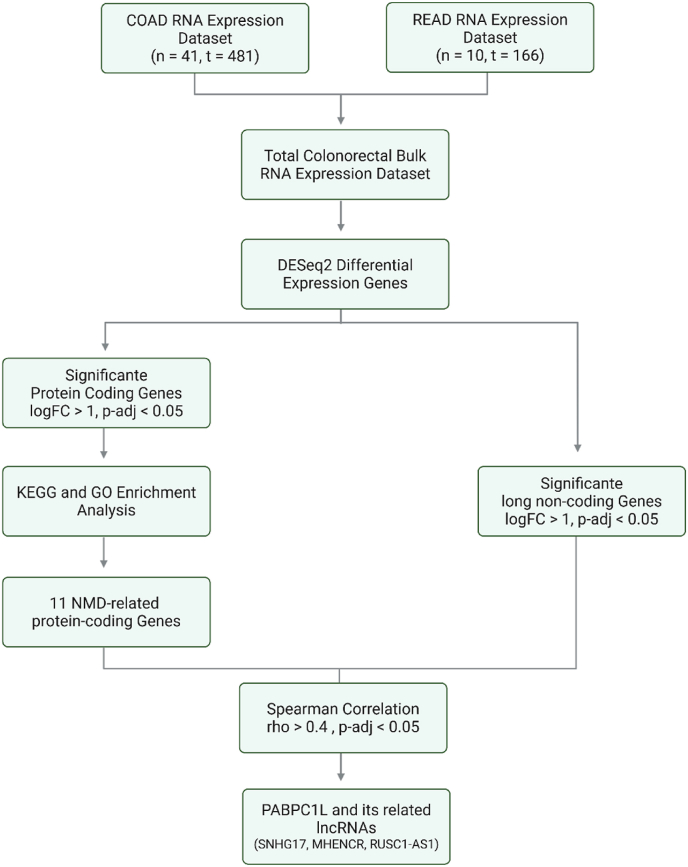
Workflow of the bioinformatics analysis performed in this study, including data preprocessing, differential expression analysis, functional enrichment, and network construction.

Finally, SNHG17, MHENCR, and RUSC1-AS1 were selected as PABPC1L-related lncRNAs for further investigation. Fig. 1 summarizes the workflow.

## 3 Patients

CRC samples and matched ANTs were retrieved from patients who had undergone a colonoscopy or tumor resection in Taleghani Hospital (2018–2022, Tehran, Iran). Subsequently, specimens were directly processed for the RNA extraction or were frozen at −80 °C until succeeding steps. Informed consent forms were signed by all patients. This study was approved by the Ethical Committee of Shahid Beheshti University of Medical Sciences (IR.SBMU.MSP.REC.1404.212) following the principles of the Declaration of Helsinki. The samples were evaluated by pathologists to approve diagnosis (CRC *vs*. non-cancerous tissue).

## 4 Expression assays

Total RNA was extracted from CRC and ANT samples using RNJia extraction kit (RN983006, ROJE Technologies Company, Iran). First strand cDNA was prepared using the SMOBIO ExcelRT™ Reverse Transcription Kit (Product code: RP1300, SMOBIO Company, Taiwan). Relative expressions of RUSC1-AS1, SNHG17, PABPC1L and SNHG1 were counted in CRC and ANT samples. Experiments were performed in the StepOnePlus Real-Time PCR System (Thermo Fisher Scientific) in duplicate. PCR reactions were prepared using RealQ Plus 2 × Master Mix Green with high ROX (AMPLIQON, Denmark). *ACTB* was selected as the housekeeping gene. Primers were acquired from the METABION Company (Germany). The nucleotide sequences of primers are shown in Table 1.

**Table 1:** Primer sequences.

Gene	Forward primer	Reverse primer
ACTB	CCTCGCCTTTGCCGATCC	CACGATGGAGGGGAAGACG
SNHG1	GCCTTCAGAGCTGAGAGGTAC	CTCAGACCTGTAACTTCAGCCTG
SNHG17	GGTCTCACCGTGAATCTCTTGG	GGTCTTGTGCAGATTGAACAGTG
RUSC1-AS1	TGTTGTCCTGGATGTCAGTGC	AGGACATGCTCATCTAGCTGG
PABPC1L	CAGAATGAACTGAAGCGCAGG	TCTGTCATCACCTTCGCACTG
MHENCR	GGACGATGGTCAGTGGACG	TCACAGAGGCCAACAGAGC

### 4.1 Statistical analysis

Statistical analyses were conducted using the Statistical Package for the Social Sciences (SPSS) version 18.0 (SPSS Inc., Chicago, IL, USA). Graphs were generated using GraphPad Prism version 9.0 for Windows (GraphPad Software, La Jolla, CA, USA). Expression levels of studied genes in CRC tissues were compared to ANTs. Expression levels in each sample were calculated using the efficiency-adjusted comparative Ct method, with ACTB as normalizer genes. Normality of the data distribution was assessed with the Shapiro-Wilk test. Differences in gene expression between tumor tissues and ANTs were evaluated using paired t-tests or the Wilcoxon matched-pairs signed rank test. The relative expression levels of genes in CRC samples were quantified using ANTs as the reference. Fold changes were calculated based on this reference, and the results are presented as mean fold change ±95 % confidence interval (CI). Receiver Operating Characteristic (ROC) curves were built to find optimal ΔCt cut-off values for each gene. The Youden index was employed to identify cut-off points that best discriminated CRC samples from ANTs. Sensitivity, specificity, accuracy, positive predictive value (PPV), negative predictive value (NPV), and area under the curve (AUC) were calculated based on these cut-offs. To further assess the discriminatory performance of gene expression as a diagnostic panel, multivariable logistic regression analysis was performed including PABPC1L, RUSC1-AS1, SNHG17, and SNHG1 as predictors, with disease status (tumor *vs*. ANT) as the dependent variable. Odds ratios (ORs) with 95 % CIs were estimated for each gene. Model fit was evaluated using the Akaike Information Criterion (AICc) and Hosmer–Lemeshow goodness-of-fit test. The classification accuracy, sensitivity, specificity, positive predictive value (PPV), and negative predictive value (NPV) were calculated based on a probability cutoff of 0.5. The overall diagnostic performance of the model was further quantified by the AUC. Correlation between gene expression levels was calculated using the Spearman correlation coefficient with the aid of GenEx software (GenEx SW, Multid Analysis AB, Gothenburg, Sweden). Expression of each gene in tumor samples (–ΔCt) was compared across dichotomous clinical factors using independent two-sided t-tests, and across variables with more than two categories using one-way ANOVA. To find the independent contributions of clinical parameters to gene expression in CRC tissues, multivariate linear regression models were constructed for each transcript using standardized –ΔCt values of PABPC1L, RUSC1-AS1, SNHG17, and SNHG1 as dependent variables. The models incorporated the following clinical predictors: sex, age, body-mass index (BMI) group, tumor location (rectum *vs*. colon), TNM stage, tumor grade (G1 *vs*. G2–G3), history of chronic disease, and family history of cancer. Regression outputs (β coefficients, standard errors, and p-values) were reported for each gene. P values were adjusted for multiple testing.

## 5 Results

### 5.1 General information

**Fig. 2:**
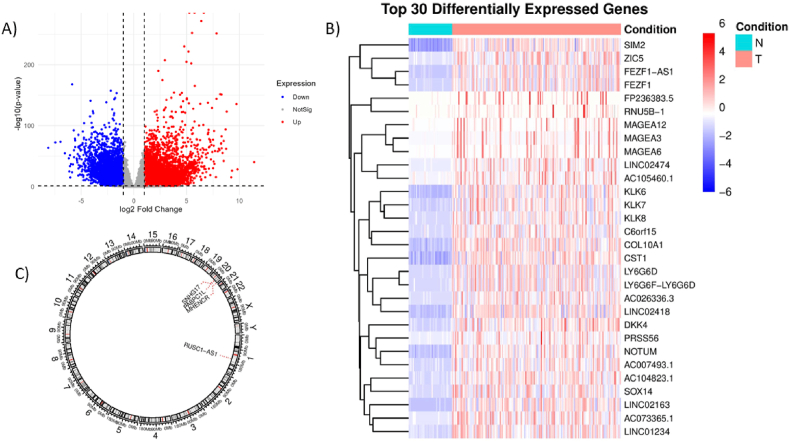
(A) Volcano plot showing differentially expressed genes (DEGs). (B) Heatmap of the top 30 DEGs. (C) Circos plot indicating chromosomal locations of the PABPC1L and its related lncRNAs.

The bioinformatics step led to identification of a number of DEGs between CRC samples and control samples. The results obtained from DESeq2, significant DEGs, results of correlation analyses and PABPC1L-related lncRNAs are shown in Supplementary file. Fig. 2 represents a summary of 30 DEGs and the chromosomal location of the selected genes for further analyses.

**Fig. 3:**
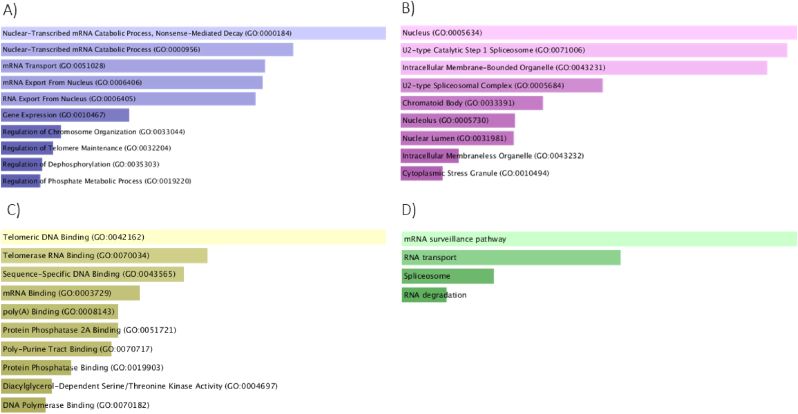
(A) Biological Process (BP), (B) Cellular Component (CC), (C) Molecular Function (MF), and (D) KEGG results based on EnrichR database.

KEGG and GO pathway analysis of the selected genes from the NMO pathways revealed related biological process, cellular component and molecular functions, which are depicted in Fig. 3.

Using Spearman's rho >0.4, and *P-adj* < 0.05 as selection criteria in the bioinformatics step, we identified three lncRNAs, namely RUSC1-AS1, MHENCR and SNHG17 as PABPC1L-related lncRNAs.

A total of 43 patients contributed paired CRC and ANT specimens and were included in the analysis (Table 2). The cohort encompassed adults across a broad age range (Mean ± SD (range): 57.57 ± 11.92 (25–87)) and included both sexes. Primary tumor sites comprised the rectum and multiple colonic segments, and most patients presented with locally advanced disease (predominantly stage II). Histologically, tumors were largely well to moderately differentiated. BMI, comorbidity burden, and family history of cancer varied across the cohort; detailed clinicopathologic characteristics and category definitions are presented in Table 2.

**Table 2:** Baseline clinical characteristics of patients with colorectal cancer (n = 43).

Variable	Category	n (percentage)
Sex	Male	28 (65.11 %)
	Female	15 (34.88 %)
BMI	<18	9 (20.93 %)
	18–25	7 (16.27 %)
	25–30	9 (20.93 %)
	30–35	16 (37.20 %)
	35–40	1 (2.32 %)
	>40	1 (2.32 %)
Tumor location	Rectum	15 (34.88 %)
	Sigmoid colon	7 (16.27 %)
	Descending colon	5 (11.62 %)
	Ascending colon	5 (11.62 %)
	Transverse colon	2 (4.65 %)
	Cecum	4 (9.30 %)
	Ileum	4 (9.30 %)
TNM stage	I	10 (23.25 %)
	II	25 (58.13 %)
	III	6 (13.95 %)
	IV	1 (2.32 %)
Tumor grade	G1 (Well differentiated)	28 (65.11 %)
	G2 (Moderately differentiated)	7 (16.27 %)
	G3 (Poorly differentiated)	5 (11.62 %)
	ND	2 (4.65 %)
History of chronic disease	Cardiopulmonary	4 (9.30 %)
	Respiratory	5 (11.62 %)
	Diabetes	3 (6.97 %)
	Hypertension	7 (16.27 %)
	None	24 (55.81 %)
Family history of cancer	Colorectal	7 (16.27 %)
	Other	9 (20.93 %)
	None	27 (62.79 %)

**Fig. 4:**
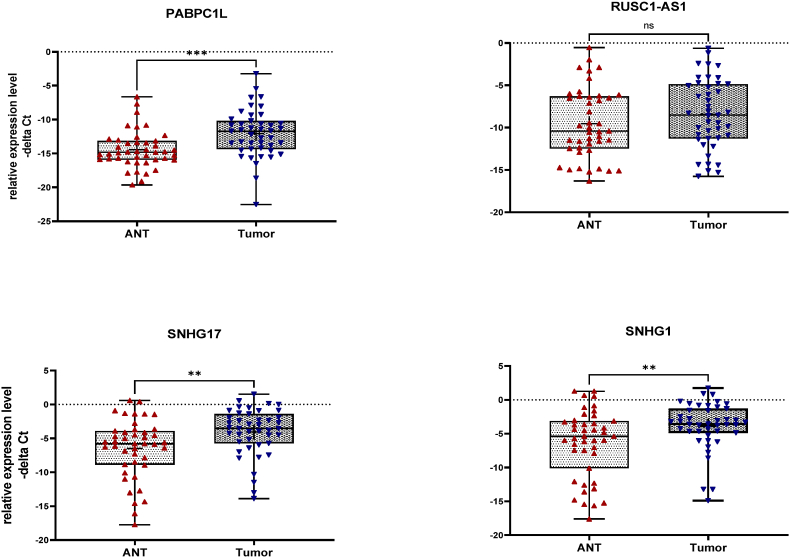
Relative expression levels of PABPC1L and three related lncRNA genes, namely RUSC1-AS1, SNHG17, and SNHG1 in colorectal tumor tissues (Tumor) and adjacent non-tumor (ANT) samples as described by –delta Ct values. The Wilcoxon matched-pairs signed rank test was used to compare expression levels between tumor tissues and ANT samples. P < 0.05 was considered statistically significant. Asterisks indicate significant difference between two mentioned groups (ns; non-significant, ∗∗P value < 0.01, ∗∗∗P value < 0.001).

Relative expression of PABPC1L, RUSC1-AS1, MHENCR, SNHG17 and SNHG1 was measured by RT-qPCR in 43 paired CRC and ANT specimens (Fig. 4, Table 3). Data are reported as −ΔCt (Ct_ACTB_ − Ct_target_) and tumor/normal fold-changes were calculated using the efficiency-corrected 2^(−ΔΔCt) method. Because the −ΔCt distributions departed from normality for all four transcripts, paired comparisons were performed using the Wilcoxon matched-pairs signed rank test. MHENCR was excluded, since its expression was not detected in a pool cDNA of ten colorectal tissue samples. Three transcripts were significantly overexpressed in tumor tissue: PABPC1L (mean fold-change ≈ 5.20, 95 % CI 2.30–11.85, P = 0.0003), SNHG17 (mean fold-change ≈ 5.46, 95 % CI 2.04–14.50, P = 0.001), and SNHG1 (mean fold-change ≈ 5.82, P = 0.0086) (Table 3). RUSC1-AS1 showed a non-significant trend toward upregulation (fold-change ≈ 2.15, 95 % CI 0.92–5.00, P = 0.07).

**Table 3:** Relative expression of PABPC1L and three lncRNAs (RUSC1-AS1, SNHG17 and SNHG1) in tumor compared to adjacent non-tumor (ANT). The expression ratio of each gene is shown as mean and 95 % CI.

Genes	Parameters	Tumor vs. ANT (43 *vs*. 43)
RUSC1-AS1	Expression ratio (95 % CI) P-value	2.15 (0.92–5) 0.07
SNHG17	Expression ratio (95 % CI) P-value	5.46 (2.04–14.5) **0.001**
PABPC1L	Expression ratio (95 % CI) P-value	5.2 (2.3–11.85) **0.0003**
SNHG1	Expression ratio (95 % CI) P-value	5.82 (1.94–4.123) **0.0086**

Fig. 4 displays the full −ΔCt distributions for each transcript (boxplots with individual datapoints), illustrating the inter-individual variability underlying these averages. Taken together, the data nominate PABPC1L, SNHG17 and SNHG1 as consistently dysregulated in CRC samples compared with matched ANTs, whereas RUSC1-AS1 demonstrated only a borderline increase and did not reach conventional statistical significance in this cohort.

Spearman rank correlation (n = 43 paired samples) revealed a reorganization of the co-expression network in tumor tissues compared with ANT (Table 4). In ANT, only modest associations were detected. SNHG17 correlated with PABPC1L (ρ = 0.40, *p* < 0.05) and PABPC1L with SNHG1 (ρ = 0.32, *p* < 0.05). By contrast, tumor specimens showed a tighter, tumor-specific module: SNHG17 correlated strongly with SNHG1 (ρ = 0.61, p < 0.01) and with PABPC1L (ρ = 0.46, *p* < 0.05), while PABPC1L and SNHG1 were also significantly associated in tumor (ρ = 0.50, p < 0.01). Importantly, RUSC1-AS1 acquired tumor-specific positive correlations with SNHG17 (ρ = 0.54, p < 0.01) and with SNHG1 (ρ = 0.45, *p* < 0.05) that were absent or weak in ANT. Collectively, these results indicate that SNHG17, PABPC1L and SNHG1 form a coordinated expression module in CRC samples and that RUSC1-AS1 is incorporated into this tumor-specific network. These findings warrant targeted functional studies to define causal relationships and shared regulatory mechanisms.

**Table 4:** Spearman's correlations between expression levels of four studied genes among tumor tissues and adjacent non-tumor (ANT) tissues. (R values are shown. ∗ Indicates P < 0.05. ∗∗indicates P < 0.001).

	SNHG17 ANT Tumor	PABPC1L ANT Tumor	SNHG1 ANT Tumor
RUSC1-AS1	0.27 0.54∗∗	0.07 0.28	−0.23 0.45∗
SNHG17		0.4∗ 0.46∗	0.21 0.61∗∗
PABPC1L			0.32∗ 0.5∗∗

**Fig. 5:**
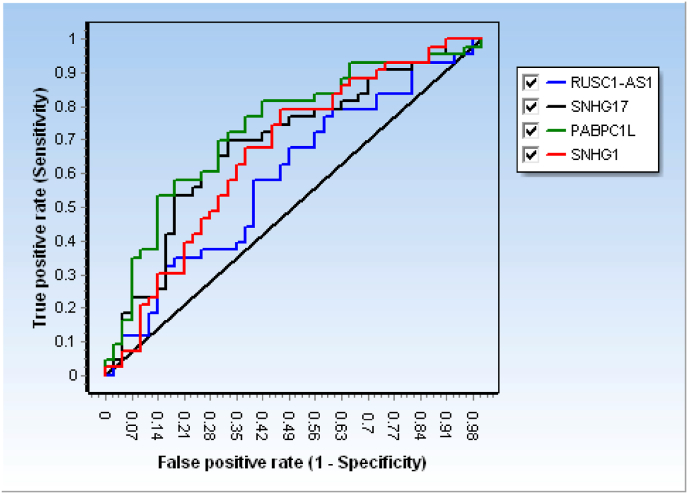
The receiver operating characteristic (ROC) curve of four studied genes for discrimination of tumor tissues from adjacent non-tumor (ANT) tissues.

ROC curve analysis was performed to evaluate the discriminatory performance of the four transcripts between tumor and ANT samples (Fig. 5, Table 5). Among them, PABPC1L demonstrated the highest diagnostic accuracy (AUC = 0.73, sensitivity 58.1 %, specificity 81.4 %, accuracy 69.8 %), followed by SNHG17 (AUC = 0.69, sensitivity 69.8 %, specificity 67.4 %, accuracy 68.6 %) and SNHG1 (AUC = 0.66, sensitivity 79.1 %, specificity 53.5 %, accuracy 66.3 %). RUSC1-AS1 showed only modest discriminatory power (AUC = 0.59, accuracy 59.3 %).

**Table 5:** Diagnostic performance of -ΔCt cut-offs for gene expression in differentiating tumor tissues from adjacent non-tumor tissues.

Gene	ΔCt Cut-off	Sensitivity (%)	Specificity (%)	Accuracy (%)	PPV	NPV	AUC
RUSC1-AS1	> −9.438	58.14 (43.4–71.6)	60.47 (45.6–73.6)	59.3	59.5	59.1	0.59 (0.47–0.71)
SNHG17	> −4.530	69.77 (54.9–81.4)	67.44 (52.5–79.5)	68.6	68.2	69.04	0.69 (0.57–0.8)
PABPC1L	> −14.67	81.4 (67.4–90.3)	58.14 (43.3–71.6)	69.8	66.03	75.8	0.73 (0.65–0.84)
SNHG1	> −5.318	79.07 (64.8–88.6)	53.49 (38.9–67.5)	66.3	63	71.8	0.66 (0.54–0.77)

Based on Youden index–derived cut-off values, tumors were more likely to be classified into the “high-expression” category for each transcript compared with ANT samples (Table 6). For example, high expression of PABPC1L was observed in 81.4 % of tumor samples versus 41.9 % of normal samples, and high SNHG1 expression in 79.1 % of tumors versus 46.5 % of ANT samples. Similarly, high SNHG17 was detected in 69.8 % of tumors compared to 32.6 % of ANT samples, while RUSC1-AS1 showed a smaller differential (58.1 % vs. 39.5 %).

**Table 6:** Classification of tumor tissues from adjacent non-tumor into high and low gene expression groups based on -ΔCt cut-offs N (%).

Groups	RUSC1-AS1 High	RUSC1-AS1 Low	SNHG17 High	SNHG17 Low	PABPC1L High	PABPC1L Low	SNHG1 High	SNHG1 Low
ANT	17 (39.52 %)	26 (60.48 %)	14 (32.55 %)	29 (67.44 %)	18 (41.86 %)	25 (58.14 %)	20 (46.5 %)	23 (53.5 %)
Tumor	25 (58.14 %)	18 (41.86 %)	30 (69.76 %)	13 (30.24 %)	35 (81.4 %)	8 (18.6 %)	34 (79.06 %)	9 (20.94 %)

Together, these findings highlight PABPC1L, SNHG17 and SNHG1 as the most promising candidate biomarkers for distinguishing colorectal tumors from adjacent non-tumor tissues, with PABPC1L showing the most favorable balance of sensitivity and specificity.

**Fig. 6:**
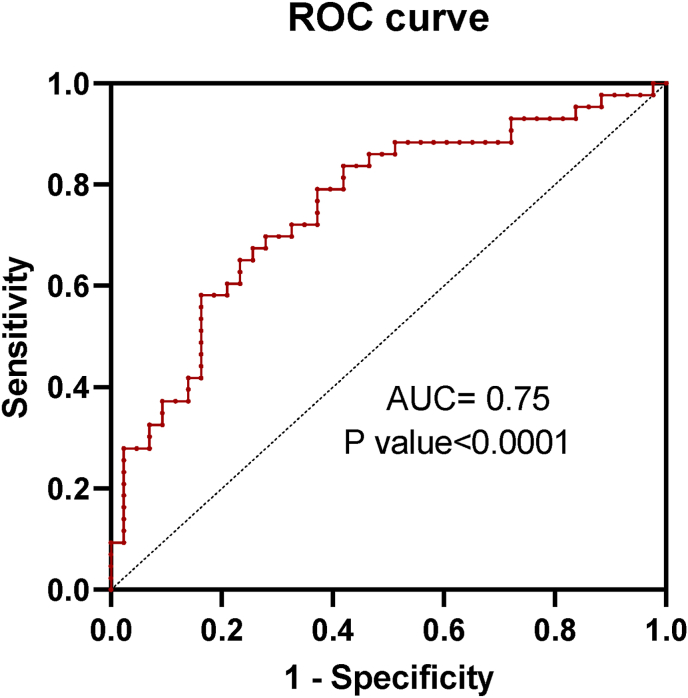
Receiver operating characteristic (ROC) curves for the multivariable logistic regression model combining PABPC1L and three lncRNAs (RUSC1-AS1, SNHG17, and SNHG1). The composite model achieved an AUC of 0. 75 (95 % CI: 0.65–0.86, *P* < 0.0001), indicating strong discriminatory ability between colorectal tumor and Adjacent non-tumor tissues.

To determine whether a multigene panel could improve discrimination between CRC tumors and matched ANTs, a multiple logistic regression model incorporating RUSC1-AS1, SNHG17, PABPC1L, and SNHG1 was constructed (Fig. 6). Among the four transcripts, only PABPC1L emerged as an independent predictor, with higher expression significantly associated with increased odds of tumor status (β = 0.172, OR = 1.19, 95 % CI 1.00–1.44, *p* < 0.05). RUSC1-AS1, SNHG17, and SNHG1 showed positive but non-significant coefficients in the multivariable context (all *p* > 0.05).

The combined four-gene model demonstrated moderate discriminatory power, yielding an AUC of 0.76 (95 % CI 0.65–0.86, *p* < 0.0001) with balanced sensitivity and specificity of 69.8 % each at the 0.5 cutoff (overall accuracy 69.8 %). Predictive values were symmetrical (PPV and NPV both 69.8 %). Model calibration was adequate, as indicated by a non-significant Hosmer–Lemeshow test (*p* = 0.47), and explained variance was modest (Tjur's R^2^ = 0.17).

Collectively, these findings suggest that while all four genes trend toward higher expression in tumor tissue, PABPC1L provides the strongest independent contribution within the multigene model, and the combined panel offers moderate diagnostic accuracy in distinguishing tumor from ANT samples.

Associations between clinical parameters and gene expression levels were evaluated using independent t-tests (for two-category variables) or one-way ANOVA (for variables with >2 categories) after clinically meaningful recoding of sparse subclasses (Table 7). Across most comparisons, no statistically significant differences in expression were detected. The only notable association was for SNHG1, which showed significantly higher expression in grade G1 tumors compared with grade G2–G3 tumors (−ΔCt −3.21 ± 3.11 vs. −6.20 ± 3.67, *p* = 0.011). No other clinicopathologic or demographic variable, including sex, BMI group, tumor location, TNM stage, chronic disease status, or family history of cancer, showed a significant relationship with the expression of RUSC1-AS1, SNHG17, PABPC1L, or SNHG1 (all p > 0.05).

**Table 7:** Association between clinical variables and tumor expression levels of PABPC1L, RUSC1-AS1, SNHG17, and SNHG1 in CRC (n = 43).

Parameters	Subclasses	Number of patients (%)	Relative expression level of RUSC1-AS1 (mean ± SD)	P-value	Relative expression level of SNHG17 (mean ± SD)	P-value	Relative expression level of PABPC1L (mean ± SD)	P-value	Relative expression level of SNHG1 (mean ± SD)	P-value
Gender	Male Female	28 (65.1) 15 (34.9)	−8.88 ± 4.18 −7.56 ± 3.96	0.32	−4.34 ± 3.6 −3.49 ± 3.4	0.45	−11.84 ± 3.84 −12.5 ± 2.93	0.57	−3.39 ± 3.18 −4.81 ± 4.17	0.22
BMI	<25 25–30 >30	16 (37.2) 9 (21) 18 (41.8)	−9.65 ± 4.1 −8.34 ± 3.53 −7.37 ± 4.3	0.8	−3.86 ± 4 −3.74 ± 2.27 −4.36 ± 3.69	0.11	−12.14 ± 4.08 −12.8 ± 3.58 −11.64 ± 3.1	0.034	−4.52 ± 3.9 −3.9 ± 2.7 −3.3 ± 3.65	0.28
Tumor location	Rectum Non-rectum	15 (34.9) 28 (65.1)	−9.38 ± 3.37 −7.91 ± 4.42	0.29	−4.67 ± 3.1 −3.71 ± 3.72	0.37	−12.42 ± 54.75 −11.88 ± 2.75	0.64	−4.41 ± 3.55 −3.6 ± 3.62	0.48
TNM/Stage	I II III & IV	10 (23.3) 25 (58.1 8 (18.6)	−7.47 ± 5.07 −8.91 ± 3.52 −8.08 ± 4.86	0.86	−3.67 ± 4.4 4.01 ± 3.64 −4.62 ± 1.73	0.3	−12.4 ± 3.42 −11.74 ± 3.63 −12.7 ± 3.68	0.5	−4.1 ± 4.04 −3.86 ± 3.87 −3.7 ± 2.03	0.09
Tumor Grade	G1 G2-G3	29 (67.4) 12 (27.6)	−8.16 ± 4.05 −9.57 ± 3.91	0.31	−3.68 ± 3.44 −5.32 ± 3.7	0.2	−11.92 ± 3.64 −13.43 ± 2.27	0.19	−3.21 ± 3.11 −6.2 ± 3.67	**0.011**
History of chronic disease	No Yes	24 (55.8) 19 (44.2)	−8.86 ± 4.12 −7.86 ± 4.13	0.43	−4.18 ± 3.38 −3.87 ± 3.74	0.78	−12.47 ± 2.91 −11.57 ± 4.22	0.41	−4.53 ± 3.6 −3.06 ± 3.47	0.18
Family history of cancer	No Yes	27 (62.8) 16 (37.2)	−7.89 ± 4.12 −9.31 ± 4.07	0.28	−3.77 ± 2.84 −4.5 ± 4.48	0.56	−11.88 ± 4.04 −12.4 ± 2.53	0.65	−3.62 ± 3.12 −4.32 ± 4.3	0.54

Multivariable linear regression was applied to assess the independent contributions of clinical covariates and co-expressed transcripts to variation in gene expression (−ΔCt values). For SNHG17 as the dependent variable, the overall model was significant (*F*(12,25) = 3.17, *p* = 0.0073, R^2^ = 0.60). Two predictors emerged: BMI was inversely associated with SNHG17 expression (β = −1.24, *p* = 0.037), and RUSC1-AS1 showed a strong positive effect (β = 0.45, *p* = 0.002), consistent with their correlation analysis. SNHG1 expression trended toward significance (β = 0.31, *p* = 0.062).

Conversely, when RUSC1-AS1 was modeled as the dependent variable, the regression was borderline significant (*F*(12,25) = 1.91, *p* = 0.083, R^2^ = 0.48). Here, SNHG17 emerged as the only independent predictor (β = 0.70, *p* = 0.002), while no clinical covariates reached significance.

For PABPC1L and SNHG1 as dependent variables, none of the clinical or molecular predictors demonstrated significant independent associations (all *p* > 0.05).

Together, these analyses indicate a robust, bidirectional coupling between SNHG17 and RUSC1-AS1, partially modulated by BMI, whereas expression variability in PABPC1L and SNHG1 was not independently explained by the tested clinical or molecular factors.

## 6 Discussion

In this paired analysis of 43 CRC patients, we found consistent upregulation of PABPC1L**,** SNHG17 and SNHG1 in tumor tissues relative to ANT samples, while RUSC1**-**AS1 showed a non-significant trend toward increased expression. Multivariable modeling indicated that PABPC1L made the strongest independent contribution to discrimination of tumor versus ANT samples within the four-gene panel, whereas the lncRNAs contributed to a coordinated tumor-specific co-expression module. These findings nominate PABPC1L and the SNHG family members as candidate components of a tissue-based diagnostic signature and motivate mechanistic follow-up. These findings are in line with the proposed role for SNHG17 in enhancement of CRC tumorigenesis and metastasis [8]. Moreover, PABPC1L encodes a cytoplasmic poly(A)-binding protein implicated in mRNA stability and translation control. A previous study has linked elevated PABPC1L to enhanced proliferation, invasion and metastasis in CRC *via* the AKT pro-oncogenic pathway [9]. Besides, PABPC1L is regarded as a metabolic and immune modulator through induction of IDO1 to promote tryptophan metabolism leading to immune suppression [10].

Members of the SNHG family are increasingly recognized as oncogenic lncRNAs that operate through diverse mechanisms, including miRNA sponging, epigenetic modulation and interaction with RNA-binding proteins. SNHG1 has been reported to promote CRC cell proliferation and invasion by sponging tumor-suppressive miRNAs, epigenetic regulation [11] and modulating SMAD2 signaling [12]. SNHG17 has been implicated in CRC progression and metastasis through SNHG17-Trim23-PES1 axis [8]. Our observation of strong tumor-specific co-expression among SNHG17, SNHG1 and PABPC1L; together with the reciprocal association between SNHG17 and RUSC1-AS1 in multivariable models suggest a model in which lncRNA networks and translation regulators act in concert to drive tumor cell phenotypes.

RUSC1-AS1 is less well characterized in CRC. In other cancers, it can function as a competing endogenous RNA (ceRNA) and modulate proliferation and apoptosis *via* miRNA-dependent axes [13,14]. The tumor-restricted emergence of positive correlations between RUSC1-AS1 and the SNHG transcripts in our dataset suggests that RUSC1-AS1 may be integrated into the tumor-specific regulatory circuitry, such as a part of a ceRNA module. However, direct functional evidence for such hypothesis is limited in CRC and requires experimental validation.

While individual transcripts provided only moderate discrimination, the combined four-gene logistic model achieved a suitable AUC value, indicating that a combined signature may improve tissue classification compared with single markers. Given the modest sample size and single-center design, these performance estimates should be regarded as preliminary. The results should be validated in independent larger cohorts (including early-stage and premalignant lesions). Moreover, the clinical applicability of these markers should be assessed in minimally invasive biospecimens. Finally, future studies are needed to evaluate whether the proposed signature adds diagnostic/prognostic value beyond traditional clinicopathologic factors and existing molecular tests.

## 7 Limitations

Our study has several limitations including the modest sample size, single-center sampling, and cross-sectional design. These limitations prevent causal inference. Although we applied efficiency-adjusted qPCR calculations, used paired statistics and examined multivariable models, potential overfitting cannot be excluded. Thus, the model would benefit from both internal validation and external replication. Additionally, no functional mechanisms were tested experimentally in the current study, thus mechanistic statements were only hypothetical. Finally, subgroup analyses were small and should be interpreted cautiously.

## 8 Future perspectives

Additional studies should validate the four-gene signature in larger, independent patient cohorts and test whether the same transcripts can be detected reliably in blood (plasma or exosomes) for a non-invasive test. Moreover, functional experiments should be designed to establish causality through knocking down or overexpressing SNHG17, SNHG1, RUSC1-AS1 and PABPC1L in CRC cell lines and measuring the effects on proliferation, migration, epithelial–mesenchymal markers and key signaling pathways.

## 9 Conclusions

Our study identified a tumor-specific expression module comprising PABPC1L**,** SNHG17 and SNHG1, with RUSC1-AS1 incorporated into the tumor co-expression network. PABPC1L was suggested to contribute independently to tumor classification in multivariable models and, together with the SNHG lncRNAs, may represent a promising candidate for validation as a diagnostic panel and for mechanistic studies for disrupting harmonized translational and lncRNA-mediated oncogenic axes in CRC.

## Declaration of competing interest

Authors declare no conflict of interests.

## Data Availability

No data was used for the research described in the article.
